# Laparoscopy versus laparotomy for the management of early stage cervical cancer

**DOI:** 10.1186/s12885-015-1818-4

**Published:** 2015-11-24

**Authors:** Yan-zhou Wang, Li Deng, Hui-cheng Xu, Yao Zhang, Zhi-qing Liang

**Affiliations:** 1 Department of Obstetrics and Gynecology, Southwest Hospital, Third Military Medical University, Chongqing, 400038 People’s Republic of China; 2 Department of Epidemiology, Clinic Epidemiology Center, Third Military Medical University, Chongqing, 400038 People’s Republic of China

**Keywords:** Laparoscopic radical hysterectomy, Abdominal radical hysterectomy, Meta-analysis, Cervical cancer

## Abstract

**Background:**

The possible advantages of laparoscopic radical hysterectomy (LRH) versus open radical hysterectomy (RH) have not been well reviewed systematically. The aim of this study was to systematically review the comparative effectiveness between LRH and RH in the treatment of cervical cancer based on the evaluation of the Perioperative outcomes, oncological clearance, complications and long-term outcomes.

**Methods:**

The systematic review was conducted by searching PubMed, MEDLINE, EMBASE, the Cochrane Library and BIOSIS databases. All original studies that compared LRH with RH were included for critical appraisal. Data were pooled and analyzed.

**Results:**

A total of twelve original studies that compared LRH (*n* = 754) with RH (*n* = 785) in patients with cervical cancer fulfilled quality criteria were selected for review and meta-analysis. LRH compared with RH was associated with a significant reduction of intraoperative blood loss (weighted mean difference = −268.4 mL (95 % CI −361.6, −175.1; *p* < 0.01), a reduced risk of postoperative complications (OR = 0.46; 95 % CI 0.34–0.63) and shorter hospital stay (weighted mean difference = −3.22 days; 95 % CI–4.21, −2.23 days; *p* < 0.01). These benefits were at the cost of longer operative time (weighted mean difference = 26.9 min (95 % CI 8.08–45.82). The rate of intraoperative complications was similar in the two groups. Lymph nodes yield and positive resection margins were similar between the two groups. There were no significant differences in 5-year overall survival (HR 0.91, 95 % CI 0.48–1.71; *p* = 0.76) and 5-year disease-free survival (hazard ratio [HR] 0.97, 95 % CI 0.56–1.68; *p* = 0.91).

**Conclusions:**

LRH shows better short term outcomes compared with RH in patients with cervical cancer. The oncologic outcome and 5-year survival were similar between the two groups.

**Electronic supplementary material:**

The online version of this article (doi:10.1186/s12885-015-1818-4) contains supplementary material, which is available to authorized users.

## Background

Cervical cancer is the fourth most common cancer in women, and the seventh overall. It accounts for 7.5 % of all female cancer deaths with approximately 266,000 deaths worldwide in 2012. Almost nine out of ten cervical cancer deaths occur in the less developed regions. In countries that do not have access to cervical cancer screening and prevention programs, cervical cancer remains the second most common type of cancer (17.8 per 100,000 women) and cause of cancer deaths (9.8 per 100,000) among all types of cancer in women [1, 2].

Radical hysterectomy with pelvic lymphadenectomy is the standard surgical treatment for patients with early stage cervical cancer [3]. Although the majority of radical hysterectomies are performed with the open technique, laparoscopic, combined laparoscopic and vaginal and robotic-assisted approaches have been used at several centers [4–7]. Compared with the abdominal radical hysterectomy, laparoscopic techniques are associated with less blood loss, shorter hospital stay, better cosmesis and faster recovery, but questions still remain about comparative effectiveness with respect to oncological clearance, complications, recurrence rates and long-term outcomes [8]. Studies comparing laparoscopy with conventional open surgery are limited by their sample sizes and are not individually powered to detect small differences in outcomes. A pooled synthesis of these studies using meta-analysis may provide further insights into the safety and comparative effectiveness of laparoscopy and conventional open surgery.

Systematic reviews and meta-analyses have shown an advantage in short-term outcomes of laparoscopic (assisted vaginal) and robotic radical hysterectomy compared with open distal radical hysterectomy [9]. Kucukmetin carried out a systematic review of randomized controlled trials (RCTs) studies that compared open and laparoscopic assisted vaginal radical hysterectomy (LAVH) in women with early cervical cancer, but found only one relevant trial which included an exceptionally small number of 13 cases. Due to the small number of cases and the short term scope of the trial, this article was unable to reach any definite conclusions regarding the relative benefits and harms of the two forms of treatment [10]. Thus far, the potential benefits and disadvantages of LRH have not been subjected to a scrupulous systematic review.

The aim of this study was to compare minimally invasive surgery, in particular, total laparoscopic radical hysterectomy (LRH) with open radical hysterectomy (RH) with respect to perioperative outcomes, oncological clearance, complications and long-term outcomes.

## Methods

### Database searching strategy

This review was conducted according to the MOOSE guidelines for systematic reviews [11]. PubMed, MEDLINE, EMBASE, the Cochrane Library and BIOSIS databases were searched for: "cervical cancer" AND "laparoscopic" AND "radical hysterectomy" along with their synonyms or abbreviations. No additional search software or special features were used. The last search update was in December, 2014. The investigators (Yanzhou Wang and Yao Zhang) independently performed the screening and article selection procedures. All articles that fulfilled the eligibility criteria were included in the systematic review. Authors were contacted by email in cases where full-text articles were not available.

### Inclusion and exclusion criteria

Studies included in this analysis must have met the following criteria: (1) adult women diagnosed with cervical cancer; (2) women who had undergone LRH versus RH as primary treatment; (3) patients who were classified as International Federation of Gynecology and Obstetrics (FIGO) stage IA1 with lymphovascular invasion to IIA. Studies were excluded from the meta-analysis if (1) radiation or concurrent chemoradiation therapy were used as primary treatment, (2) the surgical approach used was laparoscopic assisted radical vaginal hysterectomy. In the case of multiple studies with the same or overlapping data published by the same researchers, we selected the most recent study with the largest number of participants. Using these criteria, duplicate publications with derivative patients were excluded from our meta-analysis [12, 13]. One article was excluded for only including patients with stages IB2 and IIA2 and, therefore, is not comparable to this current study because this patient population includes stages IA1 through IIA2 [14].

### Data extraction

The following data were collected from each study: first author’s surname, year of publication, country, participant characteristics, study design, sample size, blood loss, transfusion rate, operative time, duration of hospital stay, intraoperative complications, postoperative complications, oncologic outcome (resection margins and mean nodal counts), recurrence rate, 5-year disease free survival (DFS) and 5-year overall survival (OS). If data could be acquired from the tabulated literature search results, they would be extracted carefully into 2 × 2 tables from all eligible publications by two independent reviewers, based on the inclusion criteria above. In the study, medians were presented instead of means. Based on these medians, the means were estimated as (low end of range + median*2 + high end of range)/4 for a sample size smaller than 25. For a sample size larger than 25, the median was used as an estimation for the mean. When only a range was provided, the standard deviations were estimated as range/4 [15]. With data regarding OS and DFS, HRs with 95 % confidence interval were not reported, data were extracted from the survival curves and mathematical HR approximations were performed using established methods [16, 17]. If data were not directly available, they would be calculated from published positive predictive values and/or negative predictive values. If there was unclear or incomplete information in the studies, the reviewers would contact the original authors for verification. Disagreements were resolved through discussion between the two reviewers.

### Quality evaluation

The NOS (Newcastle-Ottawa scale) is a tool that judges and evaluates non-randomized studies in meta-analyses [18]. The scores ranged from 0 to 9 stars. Studies with scores of 7 stars or greater were considered to be of high quality. The stars were added up to compare the quality of the study in a quantitative fashion. Two reviewers independently evaluated and cross-checked the qualities of the included studies, as well as assessed the bias of the studies. An open discussion was held to confirm the scores of those studies that caused disagreements between the reviewers.

### Statistical methods

All statistical tests were performed using the Cochrane Collaboration’s Revman5.1. Continuous data are expressed as mean differences with standard deviations (SD). Results for comparisons of dichotomous outcomes (e.g., major postoperative complications) are expressed as risk differences [or absolute risk reduction, ARR) with 95 % confidence intervals (CI)]. A meta-analysis was planned if the included studies were clinically homogeneous. Heterogeneity among studies was determined by the Chi-square-based *Q* test and the *I*^*2*^ statistics. A *p* value less than 0.05 for the *Q* test together with an *I*^*2*^ value greater than 50 % was considered a measure of severe heterogeneity. Therefore, the study was calculated using the fixed-effect model (the Mantel–Haenszel method), otherwise, the random-effects model (the DerSimonian and Laird method) was used [19]. The publication bias for each of the pooled study groups was assessed with a funnel plot. A two-tailed test was used to assess the funnel plot asymmetry; the significance was set at *p* < 0.05 level.

## Results

### Description of the studies

**Fig. 1:**
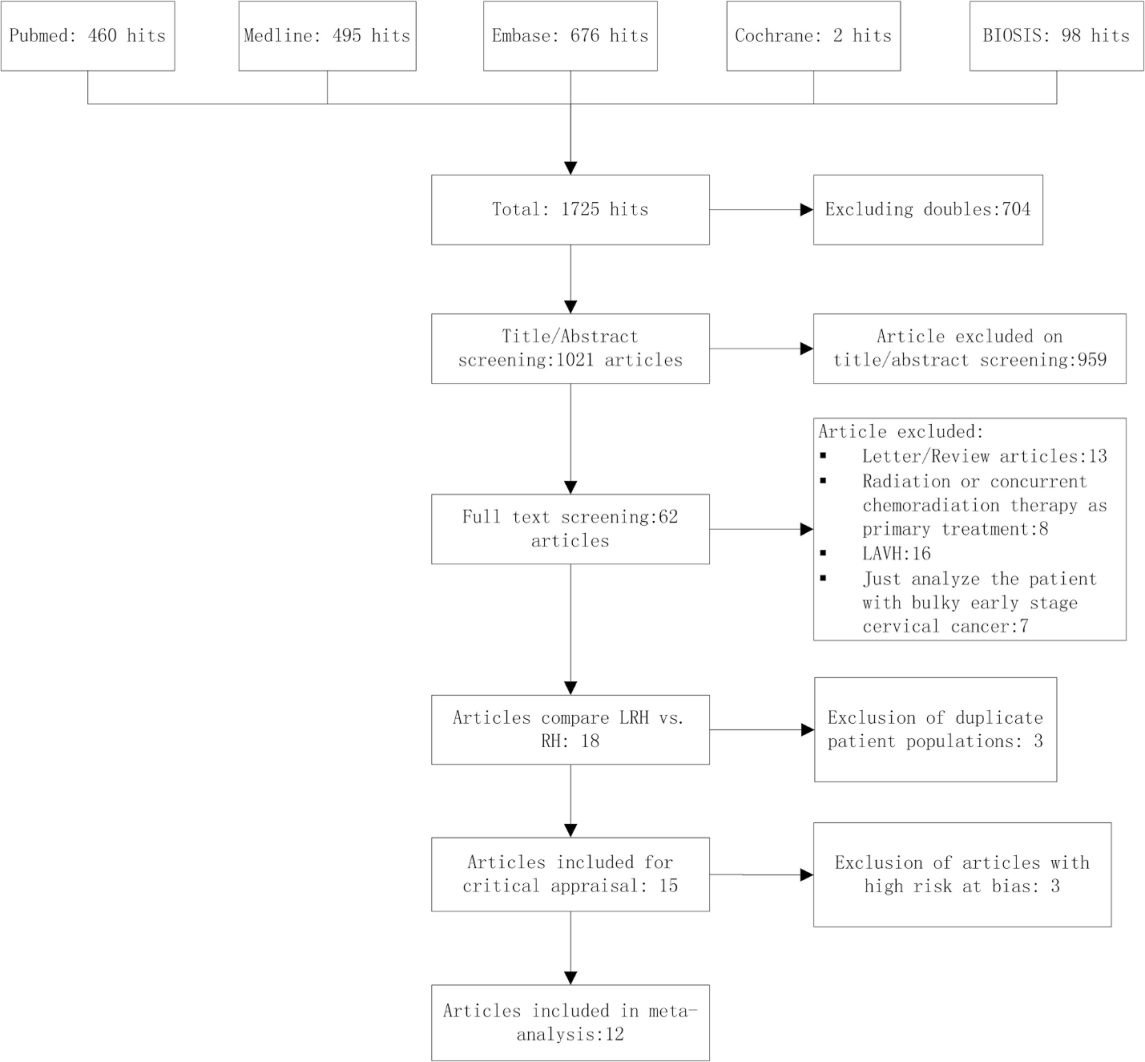
Flowchart of article screening and selection process

The selection process and result are schematically illustrated in Fig. 1. A total of 12 cohort studies were identified, all of which were accessible in full-text format. We established a database according to the information extracted from each article. Detailed characteristics of the 11 studies are listed in Table 1. A total of 754 LRH and 785 RH cases were included into our meta-analysis. Quality assessment of the studies was performed using the NOS method. The results ranged from a star rating of 6–9 (with a mean star rating of 7.75), with a higher value indicating the better methodology (Table 2).

**Table 1:** Main characteristics of 11 studies of LRH and RH

References	Design	Approach	Number	Age (years)	BMI (Kg/m2)	Tumor diameter (cm)	Stage				
							Ia1 (LVSI)	Ia2	Ib1	Ib2	IIa
Bogani et al. [31]	Propensity-matched cohort	Laparoscopic	65	48.9 ± 13.5	25.1 ± 5.2	-	-	-	-	-	-
		Open	65	50.9 ± 14	25.9 ± 6.1	-	-	-	-	-	-
Chen et al. [20]	Retrospective cohort	Laparoscopic	32	51.2 ± 11.9	23.2 ± 3.4	-	-	-	-	-	-
		Open	44	51.9 ± 11.3	24.9 ± 4.6	-	-	-	-	-	-
Ditto et al. [25]	Propensity-matched cohort	Laparoscopic	60	46 ± 12.5	24.3 ± 2.9	-	-	13	47	-	-
		Open	60	45.5 ± 15.75	24.0 ± 4.3	-	-	10	50	-	-
Frumovitz et al. [26]	Retrospective cohort	Laparoscopic	35	40.8 ± 8.75	28.1 ± 5.6	-	2	5	28	0	-
		Open	54	42.5 ± 10.25	28.2 ± 7.25	-	3	8	42	1	-
Ghezzi et al. [27]	cohort	Laparoscopic	50	47 ± 13.5	23 ± 4.4	2.6 ± 0.9	-	7	30	6	7
		Open	48	53 ± 11.8	25 ± 6.0	3.0 ± 1.0	-	2	26	13	7
Lee et al. [21]	Retrospectivecohort	Laparoscopic	24	48.4 ± 7.25	23.4 ± 3.55	-	-	5	13	2	4
		Open	48	50.2 ± 8.25	23.9 ± 4.7	-	-	10	26	4	8
Li et al. [22]	Retrospectivecohort	Laparoscopic	90	42 ± 9	-	2.8 ± 1.4	-		60	12	18
		Open	35	44 ± 11	-	2.6 ± 1.5	-		14	8	13
Lim et al. [23]	Prospectivecohort	Laparoscopic	18	47.8 ± 8.8	23.9 ± 4.4	2.9 ± 1.5	-	2	13	3	0
		Open	30	47.0 ± 8.5	22.4 ± 4	3 ± 1.2	-	1	23	4	2
Malzoni et al. [28]	Retrospectivecohort	Laparoscopic	65	40.5 ± 7.7	26.0 ± 4		5	21	39	-	-
		Open	62	42.7 ± 8.6	29.0 ± 4		3	11	48	-	-
Nam et al. [24]	Retrospectivematched cohort	Laparoscopic	263	46.4	23.2	1.8 ± 0.55	-	36	197	25	5
		Open	263	46.5	23.9	1.8 ± 0.75	-	40	194	21	8
Toptas et al. [29]	Retrospectivecohort	Laparoscopic	22	-	-	2.1 ± 1.5	-	9	13	-	-
		Open	46	-	-	2.6 ± 1.07	-	7	39		-
Zakashansky et al. [30]	Retrospectivematched cohort	Laparoscopic	30	48.3 ± 12.25	-	-	1	8	17	2	2
		Open	30	46.6 ± 11.75	-	-	1	6	19	2	2

**Table 2:** Assessment of study quality

Study	Quality indicators from Newcastle-Ottawa scale									Score
	Selection				Comparability		Exposure/outcome			
	1	2	3	4	5a	5b	6	7	8	
Bogani et al. [31]	Yes	NO	Yes	Yes	Yes	Yes	Yes	Yes	Yes	9
Chen et al. [20]	Yes	Yes	Yes	Yes	Yes	Yes	Yes	No	Yes	8
Ditto et al. [25]	Yes	Yes	Yes	Yes	Yes	Yes	Yes	Yes	Yes	9
Frumovitz et al. [26]	Yes	Yes	No	Yes	Yes	Yes	Yes	No	Yes	7
Ghezzi et al. [27]	Yes	Yes	No	Yes	Yes	Yes	Yes	No	Yes	7
Lee et al. [21]	Yes	Yes	Yes	Yes	Yes	Yes	Yes	Yes	Yes	9
Li et al. [22]	Yes	Yes	Yes	Yes	Yes	No	Yes	No	No	6
Lim et al. [23]	Yes	Yes	Yes	Yes	Yes	No	Yes	No	Yes	7
Malzoni et al. [28]	Yes	Yes	No	Yes	Yes	Yes	Yes	Yes	Yes	8
Nam et al. [24]	Yes	Yes	Yes	Yes	Yes	No	Yes	Yes	Yes	8
Toptas et al.	Yes	Yes	Yes	Yes	Yes	Yes	Yes	No	Yes	8
Zakashansky et al. [30]	Yes	Yes	Yes	Yes	Yes	Yes	Yes	No	No	7

For case–control studies, 1 indicates cases independently validated; 2 cases are consecutive or representative of population; 3 communitycontrols; 4 controls have no history of cervical cancer ;5A study controls for sex and age; 5B study controls for any additional factor(s); 6 ascertainment ofexposure by secure record or blinded interview; 7 same method of ascertainment for cases and controls; and 8 same non-response rate for casesand controls. For cohort studies, 1 indicates exposed cohort truly representative, 2 the non-exposed cohort drawn from the same community, 3ascertainment of exposure by secure record or structured interview, 4 outcome of interest was not present at start of study, 5A cohorts comparableon basis of sex and age, 5B cohorts comparable on other factor(s), 6 quality of outcome assessment, 7 follow-up long enough for outcomes tooccur; and 8 complete follow-up

The majority of the patients in 5 studies were of Asian origin and consisted of a total of 847 patients (55.0 %) [20–24]. The remaining 7 studies were European and American, comprising692 patients (39.9 %) [25–31]. Inclusion of patients was limited to those defined with FIGO stage IA1 [with lymph vascular space invasion (LVSI)] to IIA cervical cancer. The mean age ranged between 40.5 and 53.0 years. The reported BMI of Asian (means ranging between 22.4 and 24.9 kg/m^2^) was different from that of European (with means ranging between 23.0 and 29.0 kg/m^2^). The tumor diameter was similar between the two groups.

**Fig. 2:**
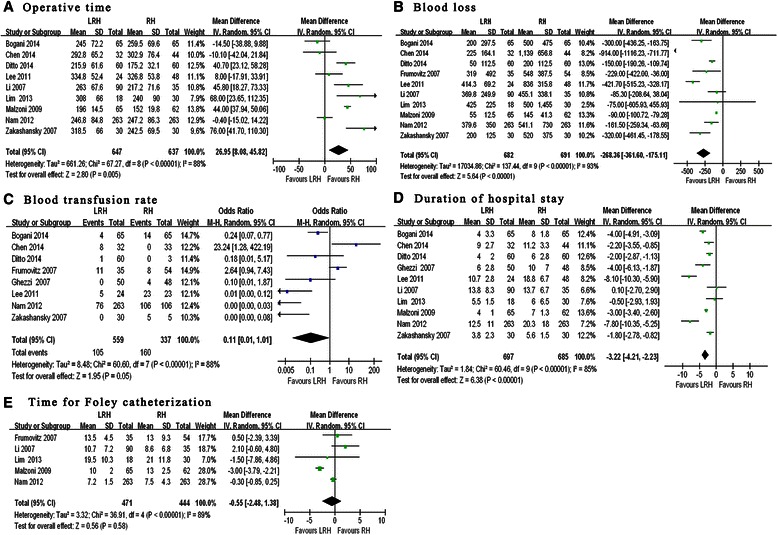
Forest plots: perioperative outcomes between LRH and RH in the treatment of cervical cancer. **a** Operative time. **b** Blood loss. **c** Blood transfusion rate. **d** Duration of hospital stay. **e** Time for Foley catheterization

The mean duration of the surgical procedure was described in the nine studies (Table 3) [20–25, 28, 30, 31]. The procedure was found to be longer for LRH in most of studies [weighted mean difference = 26.9 min (95 % CI 8.08–45.82; *p* < 0.05] (Fig. 2). The mean operative time for the laparoscopic technique was (251.5 ± 78.3) min, whereas it shortened to (240.0 ± 85.1) min for the open technique. In nine studies [20–26, 28, 30, 31], a reduction of blood loss was seen in the LRH vs. RH group [weighted mean difference = −268.4 mL (95 % CI −361.6,-175.1; *p* < 0.01] (Table 3; Fig. 2). The mean blood loss was (285.4 ± 311.1) mL in LRH compared with (524.1 ± 650.8) mL in RH, but the risk of requiring a blood transfusion was not significantly different in the laparoscopy and laparotomy groups (OR =0.11, 95 % CI: 0.01 to1.01; *p* = 0.05; Fig. 2).

**Table 3:** Study outcomes

References	Approach	Number	Operative time (min)	Blood loss (ml)	Transfusion rate (%)	Nodal counts	Duration of hospital stay	Removal of foley catheter	Surgical margins positive	5-years disease free survival, (%)	5-years overall survival, (%)
Bogani et al. [31]	Laparoscopic	65	245 ± 72.2	200 ± 297.5	4 (6)	23.2 ± 8.2	4 ± 3.3	--	--	83 %	89 %
	Open	65	259.5 ± 69.6	500 ± 475	14 (22)	27.4 ± 17.2	8 ± 1.8	--	--	80 %	83 %
Chen et al. [20]	Laparoscopic	32	292.8 ± 65.2	225.0 ± 164.1	8 (25.0)	29.7 ± 15.4	9.0 ± 2.7	--	--	--	--
	Open	44	302.9 ± 76.4	1139.0 ± 656.8	33 (75.0)	27.8 ± 11.0	11.2 ± 3.3	--	--	--	--
Ditto et al. [25]	Laparoscopic	60	215.9 ± 61.6	50 ± 112.5	1 (2)	25.4 ± 10.0	4 ± 2	--	--	--	--
	Open	60	175.2 ± 32.1	200 ± 112.5	3 (5)	34.6 ± 13.5	6 ± 2.8	--	--	--	--
Frumovitz et al. [26]	Laparoscopic	35	--	319.0 ± 492.0	11 (31.4)	--	--	13.5 ± 4.5	3 (8.6)	--	--
	Open	54	--	548.0 ± 387.5	15 (27.8)	--	--	13 ± 9.3	2 (3.7)	--	--
Ghezzi et al. [27]	Laparoscopic	50	--	--	0	21 ± 10.3	6 ± 2.8	--	3 (6.0)	--	--
	Open	48	--	--	4 (8)	23 ± 10.8	10 ± 7.0	--	3 (6.2)	--	--
Lee et al. [21]	Laparoscopic	24	334.8 ± 52.4	414.3 ± 69.2	5 (20.8)	26.3 ± 11.8	--	--	0	90.5	--
	Open	48	326.8 ± 53.8	836.0 ± 315.8	23 (47.9)	26.8 ± 13.6	--	--	0	93.3	--
Li et al. [22]	Laparoscopic	90	263.0 ± 67.6	369.8 ± 249.9	--	21.3 ± 8.4	--	10.7 ± 7.2	--	--	--
	Open	35	217.2 ± 71.6	455.1 ± 338.1	--	18.8 ± 9.5	--	8.6 ± 6.8	--	--	
Lim et al. [23]	Laparoscopic	18	308.0 ± 66.0	425 ± 225	--	17 ± 7.5	5.5 ± 1.5	19.5 ± 10.3	--	--	
	Open	30	240.0 ± 90.0	500 ± 1455	--	21.0 ± 11.8	6 ± 6.5	21.0 ± 11.8	--	--	
Malzoni et al. [28]	Laparoscopic	65	196.0 ± 14.5	55.0 ± 12.5	--	23.5 ± 5.1	--	10 ± 2	--	92.4	--
	Open	62	152.0 ± 19.8	145.0 ± 41.3	--	25.2 ± 6.2	--	13 ± 2.5	--	93.6	--
Nam et al. [24]	Laparoscopic	263	246.8 ± 84.8	379.6 ± 350.0	76 (28.9)	--	--	7.2 ± 1.5	1 (0.4)	92.8	95.2
	Open	263	247.2 ± 86.3	541.1 ± 730.0	106 (40.3)	--	--	7.5 ± 4.3	2 (0.8)	94.4	96.4
Toptas et al. 2014	Laparoscopic	22	--	--	--	--	--	--	1 (4.5)	--	--
	Open	46	--	--	--	--	--	--	1 (2.2)	--	--
Zakashansky et al. [30]	Laparoscopic	30	318.5 ± 66.0	200.0 ± 125.0	0	31.0 ± 12.8	--	--	--	--	--
	Open	30	242.5 ± 69.5	520.0 ± 375.0	5 (16.7)	21.8 ± 8.5	--	--	--	--	--

The mean hospital stay was shorter for LRH patients (weighted mean difference = −3.22 days; 95 % CI-4.21 to −2.23 days; *p* < 0.01; Fig. 2). There was no difference between the two groups in the time for Foley catheterization (weighted mean difference = −0.55 days; 95 % CI −2.48 to 1.38 days; *p* =0.58; Fig. 2).

**Fig. 3:**
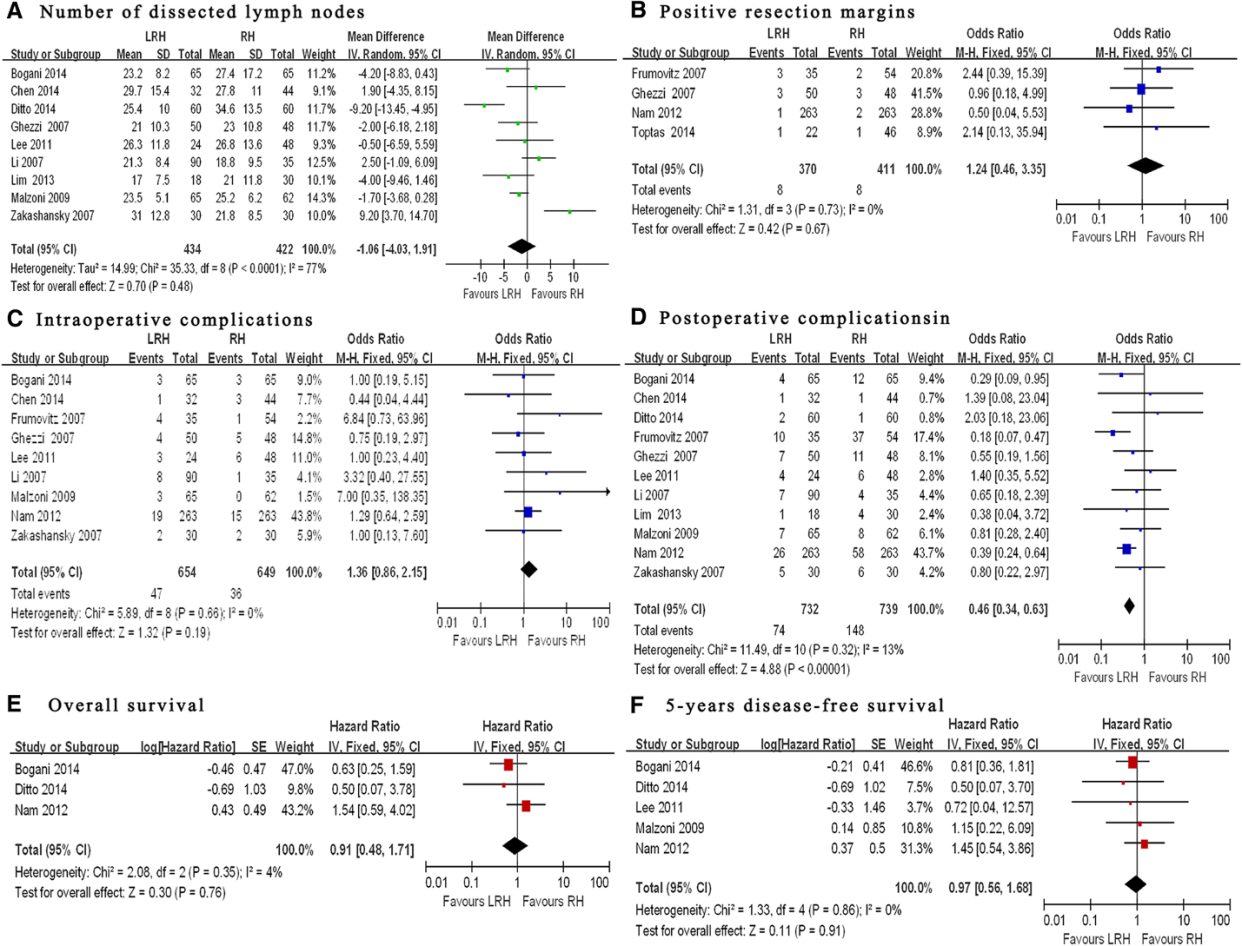
oncological clearance, complications and long-term outcomes between LRH and RH in the treatment of cervical cancer. **a** Number of dissected lymph nodes. **b** Positive resection margins. **c** Intraoperative complications. **d** Postoperative complications. **e** Overall survival, **f** 5-years disease-free survival

The number of dissected lymph nodes reported in eight studies (Table 3) [20–23, 25, 27, 28, 30, 31] showed comparable difference in both techniques (weighted mean difference = −1.06; 95 % CI −4.03 to 1.91; *p* = 0.48; Fig. 3). None of the studies reported a significant difference in positive resection margins using LRH and RH (OR = 1.24; 95 % CI 0.46–3.35; *p* = 0.67; Fig. 3).

The rate of intraoperative complications was similar in the two groups (6.4 % LRH vs. 4.9 % RH; OR = 1.36; 95 % CI 0.86–2.15; *p* = 0.19 Fig. 3; Table 4). Bladder injury occurred in 3.0 % of the LRH patients compared with 2.2 % of the RH patients (*p* = 0.309). Urethral injury was found in 1.2 % in LRH group compared with of 0.8 % in RH group (*p* = 0.425). Bowel injury was found in 0.3 % of patients in both groups (*p* = 0.992). Vascular injury occurred in 1.5 % of the LRH patients and in 1.4 % of the RH patients (*p* = 0.809) [20–22, 24, 26–28, 30, 31].

**Table 4:** Perioperative complications

References	Approach	Number	Intra-operative complication				
			Bladder injury	Urethral injury	Bowel injury	Vascular injury	Others
Bogani et al. [31]	Laparoscopic	65	1	1	0	0	1
	Open	65	0	0	0	2	1
Chen et al. [20]	Laparoscopic	32	1	0	0	0	0
	Open	44	0	2	0	1	0
Ditto et al. [25]	Laparoscopic	60	0	0	0	0	0
	Open	60	0	0	0	0	0
Frumovitz et al. [26]	Laparoscopic	35	1	0	0	3	0
	Open	54	1	0	0	0	0
Ghezzi et al. [27]	Laparoscopic	50	3	1	0	0	0
	Open	48	2	0	1	2	0
Lee et al. [21]	Laparoscopic	24	0	1	0	2	0
	Open	48	2	1	0	3	0
Li et al. [22]	Laparoscopic	90	4	0	0	4	0
	Open	35	0	0	0	1	0
Lim et al. [23]	Laparoscopic	18	0	0	0	0	0
	Open	30	0	0	0	0	0
Malzoni et al. [28]	Laparoscopic	65	1	0	0	0	2
	Open	62	0	0	0	0	0
Nam et al. [24]	Laparoscopic	263	9	6	2	2	0
	Open	263	11	3	0	0	1
Zakashansky et al. [30]	Laparoscopic	30	2	0	0	0	0
	Open	30	0	0	1	1	0
Total	Laparoscopic	732	22 (3.0)	9 (1.2)	2 (0.3)	11 (1.5)	3 (0.4)
	Open	739	16 (2.2)	6 (0.8)	2 (0.3)	10 (1.4)	2 (0.3)
*P* Value	-	-	0.309	0.425	0.992	0.809	0.6465

Postoperative complications were addressed in 11 studies (Additional file 1) [20–28, 30, 31]. The rate of postoperative surgical complications was lower for LRH versus RH groups (10.1 vs. 20.1 %; OR = 0.46; 95 % CI 0.34–0.63; *p* < 0.001; Fig. 3). The rates of wound infection (0.14 % vs. 0.94 %, *p* = 0.034), febrile morbidity (1.91 % vs. 4.74 %, *p* = 0.004), wound dehiscence (0.41 % vs. 2.30 %, *p* = 0.002) and ileus (0.82 % vs. 2.30 %, *p* = 0.022) were higher in the RH group compared to the LRH groups, where the difference was statistically significant. The rates of urinary tract infections, pelvic abscess, postoperative bleeding and ureteral stricture were also higher in the RH group, but these outcomes did not reached statistical significance. In contrast the rates of urinary tract fistula formation were higher in the LRH group without statistical significance.

Among the total 11 studies, only 3 of them reported 5-year overall survival [24, 25] and in 5 studies, 5-year disease-free survival [21, 24, 25, 28, 31]. The differences in 5-year OS (HR 0.91, 95 % CI 0.48–1.71; *p* = 0.76) and DSF (hazard ratio [HR] 0.97, 95 % CI 0.56–1.68; *p* = 0.91) were not significant (Fig. 3).

**Fig. 4:**
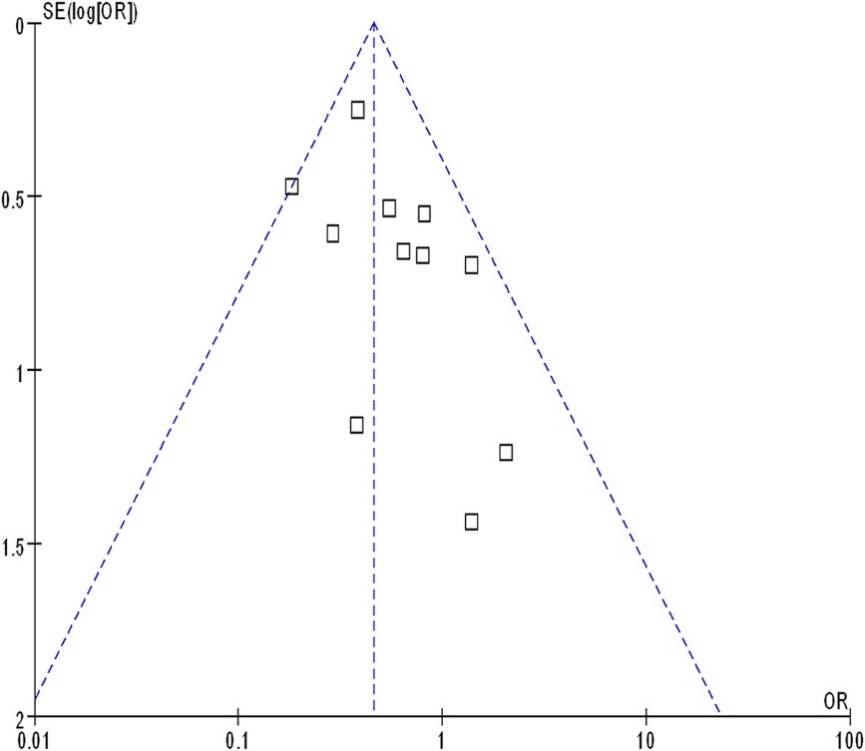
Funnel plot of studies evaluating the postoperative complications between LRH and RH groups

We used the funnel plot (Fig. 4) to examine the results of this meta-analysis. The shape of the funnel plots was nearly symmetrical on both sides of the perpendicular line (real value), indicating that the publication bias of these studies was not obvious. In order to investigate the reliability of the results, we analyzed their sensitivity. A fixed-effect and random-effect model was applied. The differences in the standardized means and the 95 % CIs between the two methods were small. Therefore, both the sensitivity and the publication bias analysis suggested that the meta-analysis results were reliable.

## Discussion

This meta-analysis was to compare LRH to RH by means of a thorough evaluation of the available evidence. All included studies were nonrandomized, nonblinded, comparative cohort studies. The studies with a high risk of bias were excluded from this meta-analysis. NOS method was applied and combined with a critical appraisal in order to provide a reliable indication of study quality. Unfortunately, the reporting of study methods and potential confounders was insufficient in several studies. Moreover, the selected studies were comparative cohort studies. Thus far, no prospective randomized controlled studies are available. Two prospective randomized controlled trials (RTCs, NCT01258413 and NCT00614211) has been designed in patients with early cervical cancer treated with laparoscopic vs. abdominal radical hysterectomy, but has not provided results yet [32 ]. We believe that this present meta-analysis gives an overview of the best available knowledge in this field.

We found that duration of the surgical procedure was longer in LRH vs. RH in the majority of the studies. We also demonstrated that patients treated with LRH recovered faster than those treated with RH in a functional manner. This is most likely contributed to by the less surgically induced trauma encountered during the procedure. The reduction of blood loss and shorter hospital stay in the LRH group partly supported this hypothesis. Besides these findings, the rates of intraoperative complications were similarly low in both groups. The most frequent intraoperative complications in the LRH group were injuries to the organs such as bladder, ureter and rectum and to great vessels. The repair of injured vessels most frequently required the conversion of laparoscopy to laparotomy. The rates of postoperative complications were significantly lower in the LRH than in the RH group. This was especially true for infectious complications, febrile morbidity, wound infection and wound dehiscence, all of which have been attributed to the laparotomy itself.

In addition, parametrial disease is an independent predictor of recurrence-free survival of cervical cancer patients. Some researchers believe that LRH is performed using an uterine manipulator, which makes the estimation of adequate vaginal resection difficult, and can potentially lead to tumor spillage, especially when the vagina is opened and the tumor surface is exposed to circulating CO_2_ [33]. Therefore, objective evidence that LRH can achieve at least the same extent of resection as in RH should be provided before using them interchangeably. Our meta-analysis did find no differences between the two types of surgery in terms of positive surgical margins and lymph nodes yield. This does suggest that laparoscopically managed patients with cervical cancer undergo a similar extent of surgery as those treated with the conventional RH. So far, no meta-analysis has summarized the long-term survival rate of cervical cancer. Only a few studies reported the survival outcomes. Our analysis showed that survival outcomes of the laparoscopic and classical open modalities were comparable, but statistical difference was hard to assess due to the insufficient data of the selected studies which included the unclear use and duration of adjuvant therapy as well as the limited number of data describing long-term survival after LRH versus RH.. It is plausible that these factors may have influenced the overall and disease-free survival of patients.

This study has some limitations that should be recognized when interpreting the results. Firstly, the cohort studies might be subjected to selection bias. Secondly, case selection may have caused the more advanced cervical cancer cases not to be considered for LRH and thirdly the selected studies in this meta-analysis can be seen as pioneer studies and therefore there is probably a learning curve associated with them that may have influenced the results in a negative manner.

## Conclusion

Our meta-analysis showed that LRH is a safe and feasible procedure to treat the early stage of cervical cancer. This was evidently supported by reduced blood loss, lower rates of postoperative complications, and faster functional recovery, with a cost of longer operative time found in LRH groups by our meta-analysis. Other outcomes including lymph nodes yield, positive resection margins, 5-year overall survival and 5-year disease-free survival by the two surgical techniques were similar. Further research in the form of prospective RCTs is warranted to evaluate long-term survival outcomes. In our opinion, future research should be directed at determining oncologic outcome, survival and quality of life in addition to the outcomes reported in this review.

## Additional file

Additional file 1: **Appendix 3.** Postoperative complications. (DOCX 30 kb)

## Abbreviations

LRH: Laparoscopic radical hysterectomy
RH: Open radical hysterectomy
RCTs: Randomized controlled trials
LAVH: Laparoscopic assisted vaginal radical hysterectomy
FIGO: International Federation of Gynecology and Obstetrics
DFS: Disease free survival
OS: Overall survival
LVSI: Lymph vascular space invasion
