# Supplementary material for: Laparoscopy versus laparotomy for the management of early stage cervical cancer
Source: BMC Cancer. 2015 Nov 24;15:928. doi: 10.1186/s12885-015-1818-4 (PMC4657298; doi:10.1186/s12885-015-1818-4)
Supplement: Additional file 1: — Appendix 3. Postoperative complications. (DOCX 30 kb) [file 12885_2015_1818_MOESM1_ESM.docx]

Additional file 1: Appendix 3 Postoperative complications

| References | Approach | n | Total | Leg lymphedema | Wound infection | Urinary tract infections | febrile morbidity | Pelvic abscess including infected lymphocele | Wound dehiscence | Incisional hernia | Bowel perforation | Postoperative bleeding | Urinary tract fistula formation | Ileus | Acute renal failure | Ischemic heart disease | Pseudomembranous colitis | Deep vein thrombosis and pulmonary embolism  DVT/PE | Brachial plexopathy | Obturator neuropathy | Ureteral stricture |
| --- | --- | --- | --- | --- | --- | --- | --- | --- | --- | --- | --- | --- | --- | --- | --- | --- | --- | --- | --- | --- | --- |
| Bogani et al 2014 | Laparoscopic | 65 | 4 | -- | -- | -- | -- | -- | 2 | -- | -- | 1 | 1 | -- | -- | -- | -- | -- | -- | -- | -- |
|  | Open | 65 | 12 | -- | -- | -- | -- | 1 | 1 | -- | 3 | 2 | 1 | 3 | 1 | -- | -- | -- | -- | -- | -- |
| Chen et al. 2014 | Laparoscopic | 32 | 1 | -- | -- | -- | -- | -- | -- | -- | -- | -- | -- | 1 | -- | -- | -- | -- | -- | -- | -- |
|  | Open | 44 | 1 | -- | 1 | -- | -- | -- | -- | -- | -- | -- | -- | -- | -- | -- | -- | -- | -- | -- | -- |
| Ditto et al. 2014 | Laparoscopic | 60 | 2 | -- | -- | -- | -- | -- | -- | -- | -- | 1 | 1 | -- | -- | -- | -- | -- | -- | -- | -- |
|  | Open | 60 | 1 | -- | -- | -- | -- | 1 | -- | -- | -- | -- | -- | -- | -- | -- | -- | -- | -- | -- | -- |
| Frumovitz et al.2007 | Laparoscopic | 35 | 9 | -- | -- | 3 | 3 | 0 | 0 | -- | -- | -- | -- | 0 | -- | -- | -- | 3 | -- | 1 | -- |
|  | Open | 54 | 37 | -- | -- | 8 | 10 | 2 | 10 | -- | -- | -- | -- | 3 | -- | -- | -- | 3 | -- | 1 | -- |
| Ghezzi et al. 2007 | Laparoscopic | 50 | 7 | -- | -- | -- | -- | 2 | -- | -- | -- | -- | 3 | 1 | -- | -- | -- | 1 | -- | -- | -- |
|  | Open | 48 | 11 | 1 | -- | -- | 3 | 2 | 1 | 1 | -- | 2 | -- | 1 | -- | -- | -- | -- | -- | -- | -- |
| Lee et al.2011 | Laparoscopic | 24 | 4 | 0 | 1 | 3 | -- | -- | -- | -- | -- | -- | -- | -- | -- | -- | -- | 0 | -- | -- | -- |
|  | Open | 48 | 6 | 1 | 1 | 3 | -- | -- | -- | -- | -- | -- | -- | -- | -- | -- | -- | 1 | -- | -- | -- |
| Li et al.2007 | Laparoscopic | 90 | 7 | -- | -- | -- | -- | 4 | 0 | -- | -- | -- | 2 | 1 | -- | -- | -- | -- | -- | -- | -- |
|  | Open | 35 | 4 | -- | -- | -- | -- | 2 | 1 | -- | -- | -- | 0 | 1 | -- | -- | -- | -- | -- | -- | -- |
| Lim et al. 2013 | Laparoscopic | 18 | 1 | -- | -- | -- | -- | -- | -- | -- | -- | -- | 1 | -- | -- | -- | -- | -- | -- | -- | -- |
|  | Open | 30 | 4 | -- | 4 | -- | -- | -- | -- | -- | -- | -- | -- | -- | -- | -- | -- | -- | -- | -- | -- |
| Malzoni et al.2009 | Laparoscopic | 65 | 7 | -- | -- | -- | 6 | -- | -- | 0 | -- | -- | 1 | -- | -- | -- | -- | -- | -- | -- | -- |
|  | Open | 62 | 8 | -- | -- | -- | 8 | -- | -- | 0 | -- | -- | 0 | -- | -- | -- | -- | -- | -- | -- | -- |
| Nam et al.2012 | Laparoscopic | 263 | 26 | -- | -- | -- | 5 | 7 | 1 | 3 | 1 | 0 | 2 | 2 | 0 | 1 | 1 | 0 | 1 | 1 | 1 |
|  | Open | 263 | 58 | -- | -- | -- | 14 | 17 | 4 | 1 | 0 | 1 | 4 | 7 | 1 | 0 | 1 | 3 | 0 | 0 | 5 |
| Zakashansky et al.2007 | Laparoscopic | 30 | 5 | -- | 0 | 0 | -- | -- | -- | -- | -- | -- | -- | 1 | -- | -- | 2 | 2 | -- | -- | -- |
|  | Open | 30 | 6 | -- | 1 | 2 | -- | -- | -- | -- | -- | -- | -- | 2 | -- | -- | 0 | 1 | -- | -- | -- |
| Total | Laparoscopic | 732 | 74 | 0 | 1 | 6 | 14 | 13 | 3 | 3 | 1 | 2 | 11 | 6 | 0 | 1 | 3 | 6 | 1 | 2 | 1 |
|  | Open | 739 | 148 | 2 | 7 | 13 | 35 | 25 | 17 | 2 | 3 | 5 | 5 | 17 | 2 | 0 | 1 | 8 | 0 | 1 | 5 |
|  | P Value |  |  | 0.1590 | 0.034 | 0.111 | 0.004 | 0.052 | 0.002 | 0.647 | 0.321 | 0.261 | 0.127 | 0.022 | 0.1590 | 0.3152 | 0.312 | 0.604 | 0.315 | 0.558 | 0.104 |
